# Congenital Internal Jugular Phlebectasia: An Anomaly Still Poorly Recognized

**DOI:** 10.1055/a-2130-3269

**Published:** 2023-08-31

**Authors:** Alessandro Raffaele, Marta Gazzaneo, Piero Romano, Maria Sole Prevedoni Gorone, Luigi Avolio

**Affiliations:** 1 Pediatric Surgery Unit, Department of Maternal and Child Health, Fondazione IRCCS Policlinico San Matteo, Pavia, Lombardia, Italy; 2 Pediatric Radiology Unit, Department of Diagnostic and Interventional Radiology and Neuroradiology, Fondazione IRCCS Policlinico San Matteo, Pavia, Lombardia, Italy

**Keywords:** jugular vein, phlebectasia, neck mass

## Abstract

Congenital internal jugular phlebectasia (CIJP) is a rare condition characterized by congenital dilatation of the vein without tortuosity that becomes more evident during straining as a lateral neck mass. CIJP often remains undiagnosed from a few months to several years after the onset of the swelling. It is frequently asymptomatic although symptomatic cases have been occasionally reported. We present the case of a healthy 7-year-old boy with a lateral neck mass, triggered by the Valsalva maneuver. Neck ultrasound (US) showed right internal jugular axial ectasia, increasing during the Valsalva maneuver; contrast computed tomography (CT) scan confirmed a fusiform dilatation of the right internal jugular vein. Due to the lack of symptoms, we treated our patient conservatively. At 5 years of follow-up, the patient is still asymptomatic, with no evidence of complications or thrombosis. Due to its self-limiting nature, treatment for asymptomatic cases of CIJP should be conservative, providing a follow-up with both clinical and US annual evaluations.

**Importance for the Pediatric Surgeon**

Even though sporadic cases of congenital internal jugular phlebectasia have been reported in the last decade, it still remains a poorly known condition, usually diagnosed from few months to several years after the onset of symptoms. Thus, it is important to consider it in the differential diagnosis of neck masses.

## Introduction

Congenital internal jugular phlebectasia (CIJP) is a rare anomaly characterized by a huge fusiform or saccular dilatation of the internal jugular vein that becomes more evident as a soft and painless lateral neck mass during straining. 1 It differs from an aneurysm since the CIJP presents as a homogeneous fusiform dilatation.

The etiology is unclear. 2 A systematic review performed on this condition 1 identified 97 articles describing 206 pediatric patients. Nonetheless, it remains a poorly known condition, usually diagnosed from a few months to several years after the onset of symptoms. 3

## Case Report

**Fig. 1:**
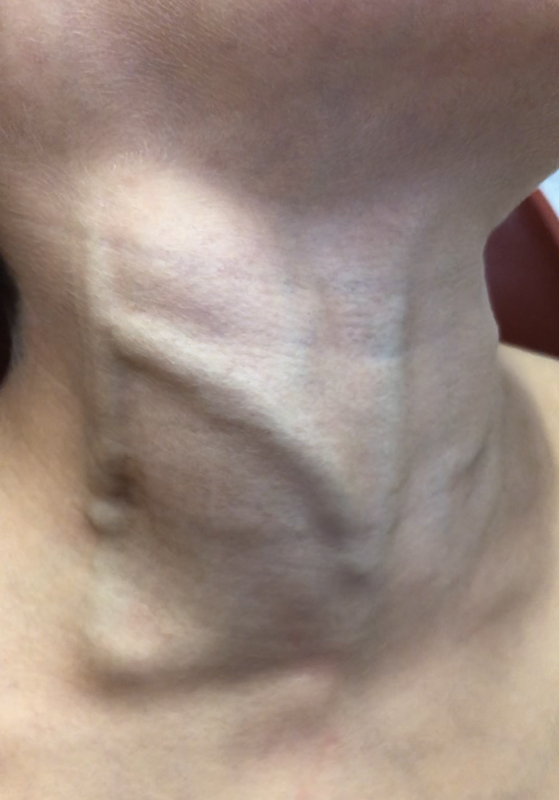
Right lateral neck mass during Valsalva maneuver.

A 7-year-old boy, weighing 21 kg, was referred to our outpatient clinic for a permanent soft swelling on the right lateral region of the neck, with no history of trauma, previous surgery, or other significant signs and symptoms ( Fig. 1 ). The mass was noticed by the parents about 4 years before the consult; however, no investigation was performed in agreement with the pediatrician. The swelling was 4 × 3 cm in size, located beneath the anterior margin of the right sternocleidomastoid muscle. It was soft and compressible, and painless with no bruit or pulsation. It became more evident under straining and was triggered by the Valsalva maneuver.

**Fig. 2:**
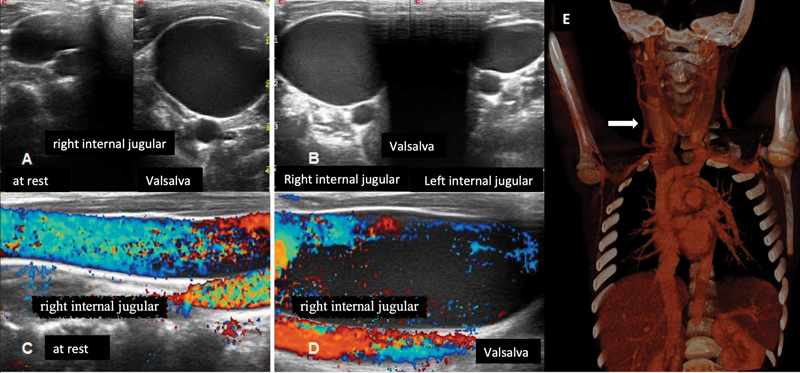
( **A** ) Ultrasound (US) of the right internal jugular vein at rest and at Valsalva maneuver; ( **B** ) right and left internal jugular veins comparative size; ( **C** ) right internal jugular vein color Doppler US demonstrating normal venous flow without thrombosis at rest and ( **D** ) under Valsalva maneuver. ( **E** ) Computed tomography (CT) contrast scan showing right internal jugular vein ectasia (shown by the arrow).

Neck US showed, at rest, right internal jugular axial 16 × 11 mm ectasia increasing to 22 × 27 mm during the Valsalva maneuver ( Fig. 2 ). The vein was detectable along its entire course, patent with regular blood flow and no thrombosis. It presented normal endoluminal valves. The contralateral internal jugular vein was normal in size and course. No other neck abnormalities were detected. Neck-thorax contrast CT scan confirmed a fusiform dilatation of the right internal jugular vein (about 22 × 17 mm at rest; Fig. 2 ), without endoluminal filling defects compatible with thrombus along its entire course. Moreover, it showed a symmetry of the subclavian veins and no arterial tortuosity or other cardiovascular anomalies.

Due to the lack of symptoms, we decided to treat the patients conservatively. At 5 years of follow-up, the patient is still asymptomatic, with no evidence of complications or thrombosis.

## Discussion

CIJP is a congenital venous anomaly resulting from structural defects in the vein walls associated with the anatomical condition, usually considered benign.

Histopathological changes can include a thinning of the muscular, elastic, and connective tissues.

It can be associated with neurofibromatosis type 1 and Ehler–Danlos syndrome. 1

The cause of CIJP is not defined. Some authors have suggested it could represent a consequence of previous injuries or medical procedures, such as neck surgeries or central venous catheterization. 1 In most cases, it remains idiopathic, as for our patient.

The right internal jugular vein is most frequently affected, probably due to anatomical features (more cranial valves, proximity of the brachiocephalic vein to the pleura, and larger diameter). 1

CIJP in pediatric patients should be distinguished from other conditions with a similar appearance in the neck as laryngocele, hemangioma, lymphatic malformation, branchial cleft cyst, and superior mediastinal masses or cysts ( Table 1 ). 4 5 6 7 8

**Table 1:** Main differential diagnoses of CIJP

Disease	Main symptoms	Site	Incidence
CIJP	Soft, compressible, painless, more evident under straining Usually asymptomatic, voice change, slight discomfort, or pain during deglutition	Lateral, anterior to the sternocleidomastoid muscle	Non Reported
Laryngocele 4	Compressible mass that increases in size with intralaryngeal pressure (external) Voice change, hoarseness, airway obstruction, hoarseness, foreign body sensation, or asymptomatic	Upper lateral	1:2.5 million
Hemangioma 5	Red or bluish soft mass Usually asymptomatic	Variable	1.64:100
Lymphatic malformation 6	Soft mass Asymptomatic or symptoms associated airways obstruction	Variable	1:250–4,000
Branchial cleft cyst 7	Cystic or tender mass Pain, dysphagia, itching skin, or asymptomatic	Lateral, anterior board of the sternocleidomastoid muscle	1:1 million
Superior mediastinal cysts and tumours 8	Airways obstruction, dysphagia, venous return obstruction, symptoms related to the underlying disease	Superior mediastinum	1:769.000–100.000

Abbreviation: CIJP, congenital internal jugular phlebectasia

CIJP is frequently asymptomatic, as in our case. Occasionally it can manifest with symptoms as change in voice, slight discomfort, or pain during deglutition. 3 9 Rarely it can lead to thrombosis or Horner's syndrome. 1 3

An accurate diagnosis can be performed with dynamic neck ultrasound, with and without Valsalva maneuver. Color Doppler allows us to study blood flow and to exclude thrombosis. Contrast CT scan or magnetic resonance (MR) angiography is recommended to confirm the diagnosis and exclude other cardiovascular anomalies. 10 11

Due to its self-limiting nature, 3 12 13 14 15 treatment for asymptomatic cases should be conservative, providing a follow-up with both clinical and US annual evaluations. When this management is considered, it should be advised to both families and patients to monitor any changes in the lesion. Incidence of thrombosis is low in the pediatric population (1.5% 1 ), but the family should be informed of the risk. Moreover, injuries should be avoided to prevent hemorrhage.

Surgical treatments have been reported for cosmetic and psychological reasons. 3

The most common procedures are represented by ligation and tapering venoplasty. 1 16 17 The ligation of the vein involves the loss of the normal venous drainage pattern on the affected side. Tapering venoplasty involves a longitudinal suture to reduce the internal diameter. Endovascular angioplasty or excision are reported as alternative surgeries.

However, several complications have been described, such as thrombosis, Horner's syndrome, or issues due to abnormal cerebral venous blood return. 13 The incidence of complications is higher in patients submitted to surgery 1 ; for this reason, we prefer conservative therapy.

Evidence on the safety of central venous catheterization in case of CIJP is missing. A case report from an adult patient suggested avoiding vein puncture due to the anomalies of the vein wall. 18 Considering the alterations of the vein wall and the pediatric age, leading to a possible increased risk of hemorrhage, it should be preferable not to perform a puncture on this vessel even in children when other possibilities exist.

Studies on long-term follow-up of pediatric patients affected by this condition are limited, but still needed to better assess the treatment of this condition and the risk of thrombosis in the long term. An international survey to collect cases and monitor clinical outcome would be desirable.
